# Clinical Applications of Artificial Intelligence, Machine Learning, and Deep Learning in the Imaging of Gliomas: A Systematic Review

**DOI:** 10.7759/cureus.19580

**Published:** 2021-11-14

**Authors:** Ayman S Alhasan

**Affiliations:** 1 Radiology, Taibah University, Medina, SAU

**Keywords:** neuro imagining, glioma classification, accuracy, neuro-oncology, artificial intelligence, deep learning

## Abstract

In neuro-oncology, magnetic resonance imaging (MRI) is a critically important, non-invasive radiologic assessment technique for brain tumor diagnosis, especially glioma. Deep learning improves MRI image characterization and interpretation through the utilization of raw imaging data and provides unprecedented enhancement of images and representation for detection and classification through deep neural networks. This systematic review and quality appraisal method aim to summarize deep learning approaches used in neuro-oncology imaging to aid healthcare professionals. Following the Preferred Reporting Items for Systematic Reviews and Meta-Analyses guidelines, a total of 20 low-risk studies on the established use of deep learning models to identify glioma genetic mutations and grading were selected, based on a Quality Assessment of Diagnostic Accuracy Studies 2 score of ≥9. The included studies provided the deep learning models used alongside their outcome measures, the number of patients, and the molecular markers for brain glioma classification. In 19 studies, the researchers determined that the deep learning model improved the clinical outcome and treatment protocol in patients with a brain tumor. In five studies, the authors determined the sensitivity of the deep learning model used, and in four studies, the authors determined the specificity of the models. Convolutional neural network models were used in 16 studies. In eight studies, the researchers examined glioma grading by using different deep learning models compared with other models. In this review, we found that deep learning models significantly improve the diagnostic and classification accuracy of brain tumors, particularly gliomas without the need for invasive methods. Most studies have presented validated results and can be used in clinical practice to improve patient care and prognosis.

## Introduction and background

Gliomas arise from precursor or glial cells and account for 27% of all tumors and 80% of major brain malignant tumors. They include glioblastoma, astrocytoma, oligodendroglioma, ependymoma, mixed glioma, malignant glioma, and not otherwise specified (NOS) and other rare histology [1]. Cellular invasion, heterogeneous angiogenesis, apoptosis, and cellular proliferation of glioma biology make its quantitative assessment complicated and significantly increase morbidity and mortality [2]. Histopathologic grading of glioma is important to plan the treatment approach, assess the response to treatment, and provide the overall prognosis. Stereotactic brain biopsy allows accurate and definitive diagnosis but is considered an invasive procedure [3].

Magnetic resonance imaging (MRI) serves as the primary contributor to brain tumor diagnosis, staging, treatment, and follow-up. The National Comprehensive Cancer Network Clinical Practice Guidelines in Oncology for Central Nervous System (CNS) Cancers recommends MRI for the evaluation of patients with a primary brain tumor and in the determination of the response to therapy [4]. Preoperative brain MRI is a useful, non-invasive imaging technique for the assessment of the histopathological grade of gliomas. Both dynamic contrast-enhanced magnetic resonance imaging (DCE-MRI) and dynamic susceptibility contrast magnetic resonance imaging (DSC-MRI) have been used prior to surgery to differentiate the grades of gliomas by using different quantitative parameters; relative cerebral blood volume (rCBV) is the most sensitive parameter [5]. In addition, computer-aided diagnosis (CAD) using intensity-invariant MRI features has been proposed to grade gliomas by using quantitative image features such as histogram moment and texture analyses, which are practical to use in the clinical setting [6]. Moreover, several approaches have been proposed for subjective visual interpretation of malignant glioma. Gutman et al. [7] developed a comprehensive subjective MRI feature called Visually AcceSAble Rembrandt Images (VASARI) to predict overall survival and correlate it with different genomic biomarkers.

Artificial intelligence (AI) is expanding rapidly and evolving in different fields including diagnostic radiology and medical imaging [8]. Machine learning (ML) is a subset of AI that allows systems to automatically learn and gain experience from existing training data and to make predictions about new data by using different algorithms and without explicit programming. Deep learning (DL) is a subfield of ML, and it uses neural networks (NN) that contain many layers to analyze different factors. In radiology and medical imaging, most ML applications rely on supervised forms comprising algorithms trained on “ground truth” labels [9]. These labels may contain different classes of diagnoses, prognoses, or classes existing in one set of images [10]. Both ML and DL methods are being used increasingly in neuro-oncological imaging. DL provides an astonishing improvement in image analysis by using raw data obtained from MRI images to automatically detect, grade, or classify gliomas. DL has become the most widely used approach within the field of ML because it can achieve outstanding results in several complex tasks, similar to and sometimes exceeding those provided by humans [11]. Multiple DL models are currently in use. These include convolutional neural network (CNN), deep Boltzmann machine (DBM), deep neural network (DNN), recurrent neural network (RNN), deep autoencoder (DA), and deep belief network (DBN) [12].

DL has the potential to detect image patterns that usually require the eyes of an experienced neuroradiologist. With the use of magnetic resonance (MR) images, DL is a noninvasive method that can rapidly identify the genetic features of glioma and make predictions regarding the treatment response and future outcome [13]. This systematic review aims to summarize ML and DL approaches used in neuro-oncological imaging to aid healthcare professionals, improve treatment outcomes, and add value to patient care.

## Review

Methods

This systematic review was registered with PROSPERO (International Prospective Register of Systematic Reviews) and was conducted according to the Preferred Reporting Items for Systematic Reviews and Meta-Analyses (PRISMA) guidelines [14]. The literature search was done with the following databases: PubMed, Medline, Cumulative Index to Nursing and Allied Health Literature (CINAHL), Web of Science, and Google Scholar. The search was done for articles in English published between 2005 and 2020, and for articles addressing the clinical application of DL in patients with glioma. The search terms included “deep learning AND glioma” OR “deep learning AND glioblastoma” OR “artificial intelligence AND glioma” OR “artificial intelligence AND glioblastoma” AND “glioma classification” AND “deep learning approaches” OR “artificial intelligence” OR “brain metastasis”.

Eligibility Criteria

All prospective and retrospective studies that examined neuro-oncology patients with glioma, glioma tumor grading, and mutations using MRI and AI, ML, or DL models as a major diagnostic tool were eligible for inclusion. The target population included patients with an established diagnosis of glioma. No restriction was applied to the patient population or age. Articles that examined radiomics and histopathological data without the use of an imaging modality were excluded. Only articles written in English were considered. All study forms were included except letters to the editor and review articles.

Data Extraction

**Figure 1:**
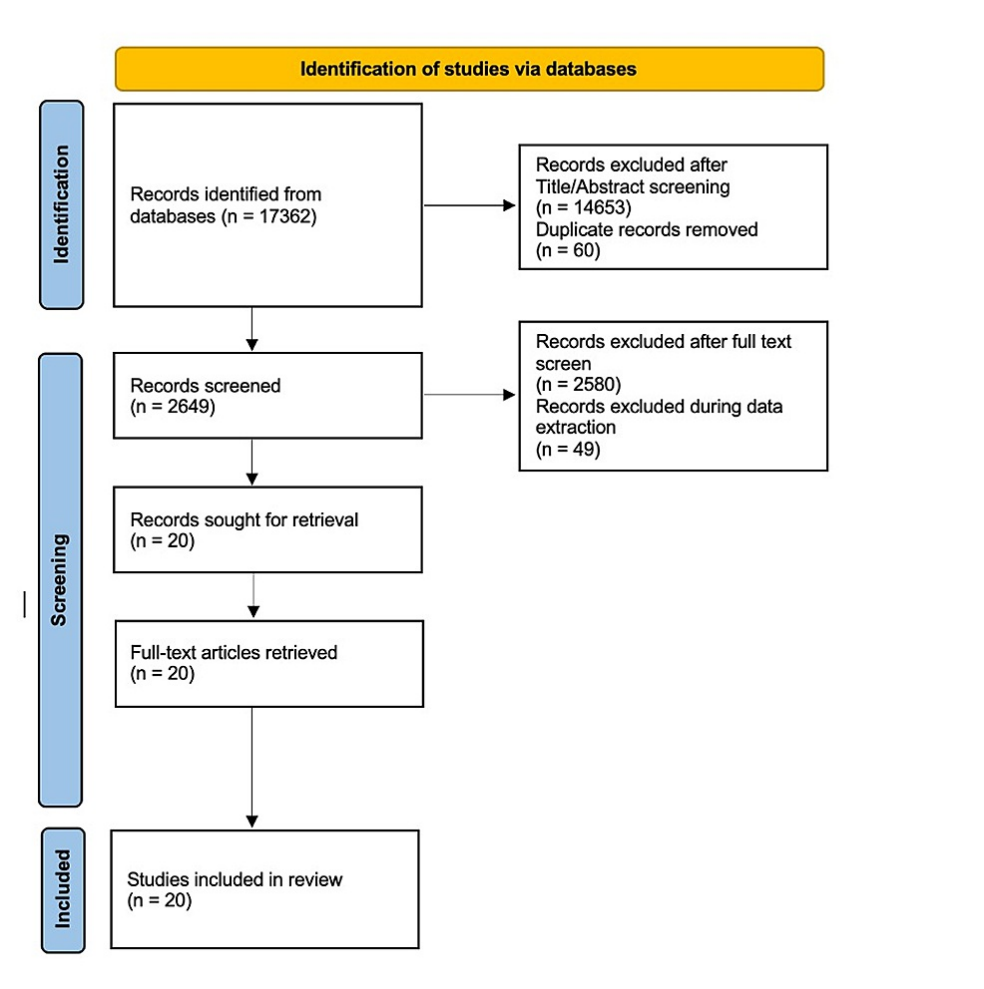
PRISMA flowchart showing the number of studies retrieved at each stage of the systematic review. PRISMA, Preferred Reporting Items for Systematic Reviews and Meta-Analyses.

Two reviewers performed the eligibility assessment of the search results by screening titles and abstracts. The review placed a limitation on the presence of a glioma tumor, the intended context for using the model, and the disease outcome of interest. Data were extracted independently by two reviewers using a predefined data extraction sheet. Furthermore, both reviewers cross-checked the extracted data and resolved any disagreements by discussion. The information for study characteristics included author(s), the purpose of the study, the number of patients or exams, the diagnostic model used, and the outcome measures including accuracy, sensitivity, specificity, the dice index, the positive predictive value (PPV), and the negative predictive value (NPV). The PRISMA flowchart showing studies retrieved at each stage of the systematic review is shown in Figure 1.

Quality Assessment of the Studies

The Quality Assessment of Diagnostic Accuracy Studies 2 (QUADAS-2) tool was used to evaluate the risk of bias, the applicability, and the quality of the studies. All studies with scores greater than 9 during the quality assessment were classified as low risk and included in this review

Data Synthesis

A single contingency table was developed to report the accuracy of each DL model used. Binary diagnostic accuracy data were extracted preferably. Contingency tables of true negatives, false negatives, true positives, and false positives were used to report sensitivity and specificity.

Results

The initial literature review produced 17,362 articles. After title and abstract screening and removal of duplicates, a total of 2,649 articles were retrieved. After the review by two independent reviewers and application of the inclusion and exclusion criteria, 2,580 articles were excluded, and an additional 49 articles were excluded during data extraction. Finally, a total of 20 articles were included in this review.

Characteristics of the Included Studies

Table 1 shows the DL models used in various studies along with their outcome measures, the number of patients or MRI scans, and the molecular markers for brain glioma classification [15-36]. In 19 studies, the researchers determined the DL model accuracy. In six studies [15-20], the authors reported the sensitivity of the DL model used, and in five studies [15,16,18-20], the authors reported the specificity of the DL models. In eight studies, the researchers examined glioma grading by using different DL models compared with other models [19,21-27].

**Table 1:** Description of deep learning models used and comparison of their outcome measures in neuro-oncology patients. 2D: two-dimensional; 2D-DOST: two-dimensional discrete orthonormal Stockwell transform; 3D: three-dimensional; ANN: artificial neural networks; AUC: area under the curve; CNN: convolutional neural network; DL: deep learning; GB: gradient boosting; HGGs: high-grade gliomas; IDH: isocitrate dehydrogenase; KNN: k-nearest neighbor; LGGs: low-grade gliomas; MGMT: O6-methylguanine-DNA methyltransferase; MLP: multilayer perceptron; NPV: negative predictive value; PPV: positive predictive value; RF: random forest; SVM: support vector machine; T2w: T2-weighted; TS: true segmentation; VGG: visual geometry group; Wndchrm: weighted neighbor distance using a compound hierarchy of algorithms representing morphology.

No.	Author	Year	Study markers	Best performing DL model used	Sample size	Other DL models used	Outcome measures	Values (DL model)
1	Akkus et al. [15]	2015	Preoperative patients with LGGs segmentation	2D and 3D segmentation	30	STAPLE TS	3D segmentation	
							Dice index	0.89
							Sensitivity	0.91
							Specificity	0.99
							2D segmentation	
							Dice index	0.9
							Sensitivity	0.92
							Specificity	0.99
2	Bangalore et al. [16]	2020	Predict IDH mutation status	T2-Net	214 patients (94 IDH mutated and 120 IDH wild-type)	T2W image only network (T2-Net)	T2-net	
							Accuracy	97.14%
							Sensitivity	0.97
							Specificity	0.98
							PPV	0.98
							NPV	0.97
							AUC	0.98
						Multi-contrast network (TS-Net)	TS-net	
							Accuracy	97.12%
							Sensitivity	0.98
							Specificity	0.97
							PPV	0.97
							NPV	0.97
							AUC	0.99
3	Díaz-Pernas et al. [17]	2021	Segmentation and classification of brain tumors (meningioma, glioma, and pituitary tumors)	Deep CNN with a multiscale approach	233	Classic ML and DL methods	Accuracy	97.30%
4	Naser et al. [18]	2020	Grade LGGs (grade II vs. III) and tumor detection by segmentation	DL model	110	Detection model	Accuracy	0.92
							Sensitivity	0.92
							Specificity	0.92
						Grading model	Accuracy	0.89
							Sensitivity	0.87
							Specificity	0.92
5	Zhuge et al. [19]	2020	Glioma classification	3DConvNet	285	2D Mask R-CNN	Accuracy	96.30%
							Sensitivity	93.50%
							Specificity	97.20%
						3DConvNet	Accuracy	97.10%
							Sensitivity	94.70%
							Specificity	96.80%
6	Nalawade et al. [20]	2019	Predict IDH mutation status	DenseNet-161	260 patients (120 HGGs and 140 LGGs)	Inception-v4	Inception-v4 slice-wise	
							Accuracy	76.10%
							Precision	59.40%
							Sensitivity	59.20%
							Specificity	84.50%
							F1 score	58.20%
							Inception-v4 subject-wise	
							Accuracy	64.20%
							Precision	65.80%
							Sensitivity	65.10%
							Specificity	65.10%
							F1 score	64.00%
						ResNet-50	ResNet-50 slice-wise	
							Accuracy	89.70%
							Precision	79.30%
							Sensitivity	81.70%
							Specificity	94.10%
							F1 score	81.30%
							ResNet-50 subject-wise	
							Accuracy	81.40%
							Precision	81.50%
							Sensitivity	81.50%
							Specificity	81.50%
							F1 score	81.40%
						DenseNet-161	DenseNet-161 slice-wise	
							Accuracy	90.50%
							Precision	79.90%
							Sensitivity	83.10%
							Specificity	94.80%
							F1 score	81.30%
							DenseNet-161 subject-wise	
							Accuracy	83.80%
							Precision	84.10%
							Sensitivity	83.50%
							Specificity	83.50%
							F1 score	83.50%
7	Gutta et al. [21]	2021	Predict glioma grade	CNN	237	CNN trained with radiomic features alone	Accuracy CNN	87%
						GB	Accuracy GB	64%
						RF	Accuracy RF	58%
						SVM	Accuracy SVM	56%
8	Latif et al. [22]	2021	Glioma tumor detection	CNN	65 patients (26 LGGs and 39 HGGs)	MLP classifier	Accuracy	98.50%
						KNN classifier	Accuracy	97.96%
						SVM classifier	Accuracy	90.04%
9	Lu et al. [23]	2020	Glioma classification	Modified ResNet50	193 cases	Modified ResNet	Accuracy	80.11%
						CNN	Accuracy	75.43%
						DenseNet	Accuracy	67.55%
						MLP	Accuracy	63.58%
						ResNet50	Accuracy	78.59%
10	Ahammed Muneer et al. [24]	2019	Glioma grade identification (LGG, oligodendroglioma, anaplastic glioma, and glioblastoma multiforme)	VGG-19 CNN		Wndchrm classifier	Accuracy	92.86%
						VGG-19	Accuracy	98.25%
11	Mzoughi et al. [25]	2020	Glioma classification into LGG and HGG	3D-CNN	284	2D-CNN	Accuracy	96.49%
12	Yang et al. [26]	2018	Glioma classification	CNN (GoogLeNet)	113 patients (53 LGGs and 61 HGGs)	AlexNet	Validation accuracy	0.866
							Test accuracy	0.855
							Test AUC	0.894
						GoogLeNet	Validation accuracy	0.867
							Test accuracy	0.909
							Test AUC	0.939
13	Khawaldeh et al. [27]	2017	Glioma classification	Modified AlexNet	109	ConvNet-3	Accuracy	85.71%
						Modified AlexNet	Accuracy	91.16%
14	Chang et al. [29]	2018	Predict IDH mutation status	Residual CNN (ResNet) with age incorporation	496	Residual CNN (ResNet) with age incorporation	Training accuracy	87.30%
							Validation accuracy	87.60%
							Testing accuracy	89.10%
						Residual CNN (ResNet) without age incorporation	Training accuracy	82.80%
							Validation accuracy	83.00%
							Testing accuracy	85.70%
15	Chang et al. [30]	2018	Predict MGMT promoter methylation status, IDH1 mutation status, and 1p/19q codeletion status	CNN	259 patients	Predict MGMT promoter methylation status	Accuracy	83.00%
							AUC	0.81
						Predict IDH1 mutation status	Accuracy	94.00%
							AUC	0.91
						1p/19q codeletion status	Accuracy	92.00%
							AUC	0.88
16	Levner et al. [32]	2009	Predict MGMT promoter methylation status		59	2D-DOST + ANN	Accuracy	87.70%
17	Korfiatis et al. [33]	2017	Predict MGMT promoter methylation status (no tumor, methylated MGMT, or non-methylated MGMT)	ResNet50	155 patients (66 methylated and 89 unmethylated tumors)	ResNet50	Test accuracy	94.40%
						ResNet34	Test accuracy	80.72%
						ResNet18	Test accuracy	76.75%
18	Ge et al. [34]	2018	Glioma classification (LGGs vs. HGGs) and 1p19q codeletion	Deep CNN	285	Deep CNN	Training accuracy	91.93%
							Validation accuracy	93.25%
							Test accuracy	90.87%
				Deep CNN	159	Deep CNN 1p19q codeletion	Training Accuracy	97.11%
							Validation accuracy	90.91%
							Test accuracy	89.39%
19	Rehman et al. [35]	2020	Classification of brain tumors (meningioma, glioma, and pituitary tumors)	CNN (fine-tuned VGG-16)	233	Fine-tuned GoogLeNet	Accuracy	98.69%
						Fine-tuned AlexNet	Accuracy	97.39%
						Fine-tuned VGG-16	Accuracy	98.04%
20	Matsui et al. [36]	2020	Prediction of LGG molecular subtype	DL model	217 patients with LGG	Prediction of LGG molecular subtype	Accuracy training	96.60%
							Accuracy test	68.70%

DL Models Used to Predict the Isocitrate Dehydrogenase Mutation Status

The isocitrate dehydrogenase (IDH) mutation status is an important marker in glioma diagnosis, prognosis, and treatment. Generally, an invasive neurosurgical procedure is required to determine the IDH mutation status. Glioblastomas with an IDH mutation have a significantly improved survival compared with IDH wild-type gliomas [28]. Out of the 20 included studies, the authors of four [16,20,29,30] used different DL models in combination with MR images to predict the IDH mutation status. Nalawade et al. [20] used T2-weighted (T2w) MRI of 120 patients with high-grade gliomas (HGGs) and 140 patients with low-grade gliomas (LGGs) and compared them between three CNN models (Inception-v4, ResNet-50, and DenseNet-161). DenseNet-161 with five-fold cross-validation was found to be the best performing model with few preprocessing steps. It attained a mean slice-wise accuracy, sensitivity, and specificity of 90.5%, 83.1%, and 94.8%, respectively, and a subject-wise accuracy, sensitivity, and specificity of 83.8%, 83.5%, and 83.5%, respectively [20]. Bangalore et al. [16] used multiparametric brain MRI of 214 patients (94 IDH mutated and 120 IDH wild-type) in combination with voxelwise DL that uses either T2w image-only network (T2-Net) or multi-contrast (T2w, fluid-attenuated inversion recovery [FLAIR], and T1 postcontrast) network (TS-net). With minimal data preprocessing, T2-Net and TS-net achieved a mean cross-validation accuracy of 97.14% and 97.12%, respectively. Chang et al. [29] used residual CNN (ResNet) to predict non-invasively the IDH status of glioma from MR images of 496 patients. Incorporation of the age at diagnosis into the model increased the accuracy from 82.8% to 87.3% for the training set, from 83.0% to 87.6% for the validation set, and from 85.7% to 89.1% for the test set. Chang et al. [30] used a CNN model in combination with MRI in 259 patients with glioma to predict the IDH1 mutation status; the model achieved an accuracy of 94.0%.

DL Models Used to Predict the MGMT Promoter Methylation Status

The O6-methylguanine-DNA methyltransferase (MGMT) gene is associated with improved prognosis and a good response to treatment with temozolomide [31]. In three studies [30,32,33], the authors evaluated different DL models to predict the MGMT gene status using MRI. Levner et al. [32] performed a texture analysis of T2, FLAIR, and T1 postcontrast MR images based on two-dimensional discrete orthonormal Stockwell transform (2D-DOST) in combination with artificial neural networks (ANN) to predict the MGMT promoter methylation status in 59 newly diagnosed patients with glioblastoma. The 2D-DOST in conjunction with ANN achieved an accuracy of 87.7%, which is comparable to that of the invasive biopsy technique (approximately 90%). Korfiatis et al. [33] compared three deep CNN architectures (ResNet50, ResNet34, and ResNet18) to evaluate their ability to predict MGMT promoter methylation. ResNet50 performed significantly better than ResNet34 and ResNet18. The test accuracy for ResNet50, ResNet34, and ResNet18 was 94.40%, 80.72%, and 76.75%, respectively. Another study by Chang et al. [30] used a CNN model and MRI of 259 patients with glioma to predict the MGMT promoter methylation status. The CNN accuracy reached 83.0%.

DL Models Used to Predict Glioma Grade or Classification

In eight studies [19,21-27], the researchers reported accurate determination of glioma grade and classification using CNN compared with other techniques. Gutta et al. [21] proposed a deep CNN model in 237 patients to predict glioma grade and compared that to ML models trained by using standard radiomic features alone. The proposed deep CNN model demonstrated an accuracy of 87.0%, outperforming the ML models using radiomic features alone. The top-performing ML model trained with radiomic features alone was gradient boosting (GB), with an average accuracy of 64.0%. Similarly, Latif et al. [22] proposed a four-step CNN technique to classify brain MR images into tumorous and non-tumorous and tested them on 65 cases by using multilayer perceptron (MLP) and achieved an average accuracy of 98.77%. Lu et al. [23] used the CNN ResNet model based on the pyramid dilated convolution to classify gliomas using MRI. The classification accuracy for the modified ResNet was 80.11% compared with 63.58%, 75.43%, 67.55%, and 78.59% for MLP, CNN, DenseNet, and traditional ResNet, respectively. Ahammed Muneer et al. [24] performed automatic glioma grade identification with the weighted neighbor distance using the compound hierarchy of algorithms representing morphology (Wndchrm) classifier and compared that to a 19-layer visual geometry group (VGG-19) deep CNN. The Wndchrm classifier showed a maximum accuracy of 92.86% compared with 98.25% for the VGG-19.

Mzoughi et al. [25] proposed a fully automatic three-dimensional (3D) CNN architecture and volumetric MRI T1 postcontrast sequence to classify brain tumors into LGGs and HGGs. The 3D-CNN model achieved an accuracy of 96.49%. Yang et al. [26] compared the performance of two trained and fine-tuned CNN models (AlexNet and GoogLeNet) in 113 patients with glioma. GoogLeNet showed better performance than AlexNet whether it was trained from scratch or pre-trained models. The validation accuracy, test accuracy, and the test area under the curve (AUC) were 86.7%, 90.9%, and 93.9%, respectively, for GoogleNet compared with 86.6%, 85.5%, and 89.4%, respectively, for AlexNet. Zhuge et al. [19] made another comparison between two CNN models (3DConvNet and two-dimensional [2D] Mask R-CNN) to grade gliomas using conventional MRI. The results showed better performance for the 3DConvNet, with a test accuracy of 0.971 compared with the 2D Mask R-CNN accuracy of 0.963. Khawaldeh et al. [27] proposed a 12-layer CNN model in combination with axial FLAIR MR images. The modified AlexNet model demonstrated a greater accuracy of 91.16% compared with 85.71% for ConvNet. Ge et al. [34] used 2D CNN to assess glioma grading by using two datasets: the first for glioma classification into LGGs and HGGs and the second to identify gliomas with/without 1p19q codeletion. The proposed model showed a high test accuracy of 90.87% for glioma classification and 89.39% for 1p19q codeletion.

DL Models Used to Detect or Classify Gliomas, Meningiomas, and Pituitary Tumors

In two studies [17,35], the researchers evaluated the detection and classification of three types of brain tumors, namely, gliomas, meningiomas, and pituitary tumors, by using deep CNN. Diaz-Pernas et al. [17] presented a fully automatic segmentation and classification model using deep CNN with a multiscale approach and achieved a classification accuracy of 0.973. Rehman et al. [35] evaluated three CNN architectures with data augmentation techniques (AlexNet, GoogLeNet, and VGG-16). The VGG-16 fine-tuned architecture achieved the highest classification and detection accuracy of 98.69%.

DL Models Used for Segmentation of LGGs

MRI segmentation of LGGs is challenging because they rarely enhance after administration of gadolinium. In three studies [15,18,36], the authors investigated the segmentation of LGGs by using deep learning models. Akkus et al. [15] proposed a semi-automated segmentation process using only T2w and optionally postcontrast T1-weighted images and compared that to manual segmentation by three experts. Matsui et al. [36] developed a DL model that was able to predict the molecular subtypes of LGGs by using three different imaging modalities: positron emission tomography (PET), MRI, and computed tomography (CT). The performance of the model combining the three modalities had an accuracy of 96.6% for the training set and 68.7% for the test set. Naser and Deen [18] used T1-precontrast, FLAIR, and T1-postcontrast MR images to grade and segment LGGs. The tumor detection model achieved an accuracy of 0.92 while the grading model achieved an accuracy of 0.89.

Discussion

This systematic review summarizes the DL models used to classify and grade gliomas as well as the status of different molecular biomarkers. In three studies [16,20,29], the authors discussed the role of DNN in IDH1 mutation detection. In two studies [32,33], the authors discussed the MGMT promoter methylation mutation status with different DNN. Ge et al. [34] discussed 1p19q codeletion mutation along with glioma grading and Chang et al. [30] classified gliomas based on their genetic category (MGMT promoter methylation status, IDH1 mutation status, and 1p19q codeletion) using CNN architecture. Considering glioma classification, in several studies [19,21-27], the researchers discussed grading gliomas into low and high grades to adapt the treatment approach appropriately. The authors of two studies [17,35] evaluated the diagnostic accuracy of DNN through cross-validation. In three studies [15,18,36], the researchers specifically focused on LGG classification through CNN models to identify the most accurate and sensitive model for preoperative diagnosis.

In this review, the authors of six studies [16,19,26,27,33,35] compared different DNN to establish an accurate and effective model. Levner et al. [32] used L1-regularized NN and quantitative assessment of tumor texture to predict the MGMT promoter methylation status in 59 newly diagnosed patients with glioblastoma multiforme (GBM). Korfiatis et al. [33] tested three different residual CNN models. ResNet50 (accuracy 94.90%) outperformed ResNet34 (80.72%) and ResNet18 (76.75%). Deepak and Sarath [37] evaluated a DNN model (ResNet) in brain tumor classification using MRI and attained excellent processing with an accuracy of 98.3%. When Liu et al. [38] used the G-ResNet model (global average pooling residual network) to classify brain tumors using ResNet34, they attained 95% accuracy, which is significantly better than the previously used DNN models. Ghosal et al. [39] used the SE-ResNet-101 model to classify three brain tumors (glioma, meningioma, and pituitary tumors) without data augmentation and the proposed CNN attained an accuracy of 89.93%. In two studies [17,35], the authors examined multiple brain tumor classification by using deep CNN models. This approach produced a tumor classification accuracy of 97.3%, higher than the classic ML models [17]. Comparison between DNN (GoogLeNet, AlexNet, and VGG16) was performed and the fine-tuned VGG-16 demonstrated the highest accuracy of 98.69%. Ghosh et al. [40] used improved U-Net with VGG16 architecture cross-validated in patients from The Cancer Genome Atlas Low Grade Glioma (TCGA-LGG) dataset for tumor segmentation. The accuracy was 99.75% for the improved U-Net model, outperforming the basic U-Net model, which had an accuracy of 99.4%.

On the other hand, NN, as identified in other studies, utilizes energy to activate neurons. Only a small number of neurons are active throughout the thought process with the human brain, whereas the neurons that will be used in the future are temporarily unregulated until they are required. Single-task allocation for subsequent neurons reduces communication costs. It is anticipated that ANN will be developed in the future to help complete more multifaceted tasks.

DL approaches have a wider application in the clinical field. In this regard, use-cases of DL networks are employed for conducting medical diagnoses. As discussed previously, this process encompasses prediction, segmentation, classification, and detection. The findings of the reviewed studies show that DL methods can be dominant with respect to other high-performing algorithms. Thereby, it is safe to assume that DL will endure and continue to expand its offerings. The future progression of DL shows more potential in different fields of medicine, specifically in the realm of medical diagnosis. On the other hand, it is currently not clear whether DL can replace the role of clinicians or doctors in medical diagnosis. In this regard, DL can offer better support for professionals in the clinical field. All predictors show a broader aspect of AI and DL in different fields. Conventional approaches to different similarity measures are ineffectual compared with DL. Based on such outcomes, it has been recommended that DNN and DL will succeed, and they will be explored for a myriad of other uses in the near future.

AI could revolutionize all stages of the pathway by which patients with gliomas are managed: the postoperative acute phase; outpatient and oncological care preoperative screening, treatment planning, and diagnosis; and intraoperative tissue analysis and intraoperative workflow analysis. In addition, AI could change how national guidelines and policies are formed and help research into brain tumors as well as therapeutics. In this regard, AI could enhance clinical findings for patients in the future. Several obstacles exist for the development of AI in the field of brain tumors. The collaboration will be fundamental to developing clinically applicable AI as the field quickly diversifies. Such collaboration must emphasize the progression of databases and sources that might be utilized to train additional AI models.

This systematic review represents a simple, precise, and objective article that should contribute to the existing body of literature concerning the use of DL in neuro-oncology. The research outcomes of the included studies offer adequate information and insight into the applications of DL and AI to detect, classify, segment, and diagnose different impairments and diseases in certain anatomical realms of interest. The most important issue regarding ML and clinical medicine that should be taken into consideration is that most of the papers did not perform validation. They either developed models or performed cross-validation. According to the guidelines for developing and reporting ML predictive models in biomedical research, validation is necessary [41]. The application of AI and DL will continue to develop beyond the significant findings that have been shown in imaging gliomas. This may elevate the quality and efficiency of health care in the long term and, therefore, reduce the risk of late diagnosis of extreme diseases. On the other hand, there is still a long road before objective NNs are used widely in medical diagnosis. Finally, it is anticipated that AI will increase the combination of complex reasoning and representation learning in neuroradiological and neurosurgical practice [42,43].

## Conclusions

ML and DL models incorporating MRI have been evaluated extensively. They have a significant value in improving the diagnostic and classification accuracy of brain tumors, especially gliomas, without the need for invasive methods. Most studies have presented validated results and can be used in clinical practice to improve patient care and prognosis. Open access to such algorithms is essential to support broader technological progression because ML algorithms have become more advanced. Clinical trials must follow reporting guidelines to ensure robust evidence is collected and to reduce biases as AI platforms associated with brain tumor surgery develop. There remain valid issues about the further implementation of machines in modern neurosurgery when AI promises to enhance patient management. Enhancements in patient findings might be challenged by job replacement, unique neglect, and physician deskilling. Clinician acceptability and stringent patient approval must be considered in the future to ensure that the potential of AI, ML, and DL does not lead to unidentified adverse outcomes.
